# Peri- and post-operative management of the “high-risk” surgical patient. an audit of practice in a large district general hospital

**DOI:** 10.1186/2197-425X-3-S1-A241

**Published:** 2015-10-01

**Authors:** L Winslow, L Bridge

**Affiliations:** Intensive Care Unit, Whiston Hospital, Prescot, United Kingdom

## Intr

In 2011, the National Confidential Enquiry into Patient Outcome and Death (NCEPOD) published a report, “Knowing the Risk,” [1] which reviewed data regarding peri-operative management of the high risk surgical population and subsequently produced recommendations in aim to improve outcomes in this patient population.

Of particular focus, recommendations included; clear documentation of operative mortality risk, to use peri-operative cardiac output monitoring with CardioQ technology, as supported by NICE Medical Technology guidance 3 [2] and to provide higher-level care, with Critical Care input, post-operatively.

## Aim

This study aimed to audit clinical anaesthetic practice at a large district general hospital (DGH) against those recommendations outlined in the NCEPOD 2011 report, regarding the management of high risk surgical patients.

## Methods

Case notes were used for retrospective data collection. Patients undergoing emergency surgery between January-March 2013 and January-March 2014 were identified. From these, high-risk patients were selected for inclusion in the audit. For the purpose of this audit, high-risk patients were defined as those older than 65 years and undergoing emergency laparotomy. Data included demographic details, documentation of risk, peri-operative haemodynamic monitoring used, 30-day survival and involvement of Critical Care post-operatively. These data were collated and compared between 2013 and 2014, identifying any interval improvement, and with the national prospective data outlined in the NCEPOD report.

## Results

33 and 23 patients were identified as undergoing high-risk surgery in 2013 and 2014, respectively.

NCEPOD data identified arterial lines; central lines and cardiac output monitoring were used in 27%, 14% and 5%, in high-risk patients, respectively. In this audit, these figures were 50%, 36.1% and 2.8%, in 2013, and 60.9%, 26.1% and 34.8%, in 2014, respectively.

In this audit, 69% and 65% of “high-risk” patients were referred to higher-level care in 2013 and 2014, respectively. Of these, 85% (2013) and 90% (2014) had a clearly documented plan from higher-level care, whether that be admission to HDU or outreach plan. The overall admission rates to higher level care were 47.2% and 65.2% in 2013 and 2014, respectively. This is comparable to the 22.1% seen in the NCEPOD data.

## Conclusions

Comparison between 2013 and 2014 data suggests peri-operative care is improving year-on-year after publication of this NCEPOD report. However, there is a need for further improvement in peri-operative care to fully comply with NCEPOD recommendations.
